# Ca^2+^-dependent phosphoregulation of the plasma membrane Ca^2+^-ATPase ACA8 modulates stimulus-induced calcium signatures

**DOI:** 10.1093/jxb/erx162

**Published:** 2017-05-20

**Authors:** Alex Costa, Laura Luoni, Claudia Adriana Marrano, Kenji Hashimoto, Philipp Köster, Sonia Giacometti, Maria Ida De Michelis, Jörg Kudla, Maria Cristina Bonza

**Affiliations:** 1 Department of Biosciences, University of Milan, Milan, Italy; 2 Institute of Biophysics, Consiglio Nazionale delle Ricerche, Milan, Italy; 3 Institut für Biologie und Biotechnologie der Pflanzen, Universität Münster, Münster, Germany

**Keywords:** *Arabidopsis thaliana*, calcineurin B-like protein, Ca^2+^ signature, CBL-interacting protein kinases, plasma membrane Ca^2+^-ATPase, phosphorylation

## Abstract

Ca^2+^ signals are transient, hence, upon a stimulus-induced increase in cytosolic Ca^2+^ concentration, cells have to re-establish resting Ca^2+^ levels. Ca^2+^ extrusion is operated by a wealth of transporters, such as Ca^2+^ pumps and Ca^2+^/H^+^ antiporters, which often require a rise in Ca^2+^ concentration to be activated. Here, we report a regulatory fine-tuning mechanism of the *Arabidopsis thaliana* plasma membrane-localized Ca^2+^-ATPase isoform ACA8 that is mediated by calcineurin B-like protein (CBL) and CBL-interacting protein kinase (CIPK) complexes. We show that two CIPKs (CIPK9 and CIPK14) are able to interact with ACA8 *in vivo* and phosphorylate it *in vitro*. Transient co-overexpression of ACA8 with CIPK9 and the plasma membrane Ca^2+^ sensor CBL1 in tobacco leaf cells influences nuclear Ca^2+^ dynamics, specifically reducing the height of the second peak of the wound-induced Ca^2+^ transient. Stimulus-induced Ca^2+^ transients in mature leaves and seedlings of an *aca8* T-DNA insertion line exhibit altered dynamics when compared with the wild type. Altogether our results identify ACA8 as a prominent *in vivo* regulator of cellular Ca^2+^ dynamics and reveal the existence of a Ca^2+^-dependent CBL–CIPK-mediated regulatory feedback mechanism, which crucially functions in the termination of Ca^2+^ signals.

## Teaser

The plasma membrane Ca^2+^-ATPase ACA8 is a novel target of Ca^2+^-dependent CIPK–CBL complexes which tunes the pump activity affecting a stimulus-induced cytosolic Ca^2+^ transient *in planta*.

## Introduction

In all eukaryotes, calcium ions are involved in many biological processes, regulating a variety of essential cell functions. Cytosolic Ca^2+^ acts as a second messenger mediating cellular signalling in the transduction of a large range of endogenous and environmental signals (Berridge *et al.*, 2000; Sanders *et al.*, 2002; White and Broadley, 2003; Dodd *et al.*, 2010; Kudla *et al.*, 2010; Steinhorst and Kudla, 2013).

To avoid the toxicity of Ca^2+^, a low cytosolic concentration is maintained in resting conditions (<0.1 μM; Berridge *et al.*, 2000; Sanders *et al.*, 2002). The level of cytosolic Ca^2+^ represents the balance between passive ion influx from the outside and/or intracellular compartments introducing Ca^2+^ into the cytoplasm, cytoplasmatic buffering, and active ion removal from the cytoplasm. Perception of signals generates elevations in cytosolic Ca^2+^ concentration triggered by the opening of Ca^2+^ channels, while active Ca^2+^ transporters (Ca^2+^/H^+^ exchangers and Ca^2+^ pumps) determine the recovery of the basal pre-stimulus cytosolic free Ca^2+^ concentration ([Ca^2+^]_cyt_) (Sanders *et al.*, 2002; Berridge *et al.*, 2003; McAinsh and Pittman, 2009; Dodd *et al.*, 2010; Kudla *et al.*, 2010; Steinhorst and Kudla, 2013). Physical parameters of Ca^2+^ spikes such as amplitude, frequency, localization, and duration appear to be stimulus specific and are likely to encode information governing appropriate downstream responses. Depending on the stimulus, these dynamics can display the form of a single transient or of repetitive Ca^2+^ oscillations, and are commonly referred to as Ca^2+^ signatures (Webb *et al.*, 1996; McAinsh and Pittman, 2009; Kudla *et al.*, 2010; Steinhorst and Kudla, 2013). Each cell type is equipped with a peculiar combination of components to generate a Ca^2+^ signalling system with different spatial and temporal properties depending on the nature of the stimulus. Consequently, the generation of a Ca^2+^ signature is the result of the co-ordinated action of both Ca^2+^ influx and Ca^2+^ efflux systems localized at both the plasma membrane (PM) and membranes of the different subcellular compartments. Ca^2+^ sensor proteins decode and relay the information contained in the Ca^2+^ signature into phosphorylation cascades, modification of enzymes activity, and alteration of gene expression, thus allowing the organism to set up the proper cellular response (DeFalco *et al*., 2010; Kudla *et al.*, 2010; Hashimoto and Kudla, 2011; Spalding and Harper, 2011; Batistič and Kudla, 2012). Although many components of the Ca^2+^ signalling network have been functionally characterized, the mechanisms by which they are regulated to shape complex Ca^2+^ signatures are only beginning to be elucidated. Fine-tuning of both influx and efflux Ca^2+^ transport systems in response to different signals probably represents a crucial feature of Ca^2+^-mediated signal transduction. Ca^2+^ pumps and Ca^2+^/H^+^ exchangers are likely to play a role in determining the magnitude and duration of the Ca^2+^ spike as well as in returning [Ca^2+^]_cyt_ to resting levels, thus contributing to termination of the stimulus and modulation of specific Ca^2+^ dynamics (McAinsh and Pittman, 2009; Dodd *et al.*, 2010; Kudla *et al.*, 2010; Steinhorst and Kudla, 2013). The presence of multiple isoforms of these transporters and the complexity of their regulation mechanisms are also consistent with their putative function as modulators of the Ca^2+^ activities. Activation of distinct Ca^2+^ efflux transporters or the differential regulation of an individual efflux transporter might give rise to different Ca^2+^ transport kinetics which would return [Ca^2+^]_cyt_ to the resting value at different rates. This mechanism would therefore be able to impact on the spatio-temporal characteristics of the Ca^2+^ signature.

Ca^2+^ pumps are high-affinity, low turnover pumps that mediate Ca^2+^ extrusion from the cytoplasm and have been found at the PM and at the membranes of the main intracellular Ca^2+^ stores (Geisler *et al.*, 2000; Brini and Carafoli, 2009; Bonza and De Michelis, 2011, Brini *et al.*, 2013). Available evidence supports their involvement in fundamental processes such as development and hormonal regulation. In mammals, the function of these pumps is essential so that pump defects produce severe alterations of Ca^2+^ homeostasis that cause disease processes (Brini and Carafoli, 2009; Brini *et al.*, 2013; Lopreiato *et al.*, 2014) while, in plants, the physiological role of Ca^2+^-ATPases is associated with fundamental functions such as the response to environmental stimuli (Bonza and De Michelis, 2011). Manipulation of different plant and animal Ca^2+^ pumps perturbs cytosolic Ca^2+^ homeostasis and Ca^2+^ oscillations, and alters downstream responses. Most of these data come from overexpression of transporters in ‘single-cell’ heterologous systems such as oocytes, HeLa cells, or yeast cells (Lechleiter *et al.*, 1998; Zhao *et al.*, 2001; Kamagate *et al.*, 2002; Rimessi *et al.*, 2005; Anil *et al.*, 2008). Genetic evidence using loss-of-function plant mutants also indicates that the cytosolic Ca^2+^ dynamics can be controlled by Ca^2+^ efflux systems (Qudeimat *et al.*, 2008; Zhu *et al.*, 2010; Frei dit Frey *et al.*, 2012). In addition, pharmacological inhibition of different Ca^2+^-ATPase classes in *Arabidopsis thaliana* seedlings suggests that these pumps are involved in the regulation of both cytosolic and endoplasmic reticulum Ca^2+^ homeostasis (Bonza *et al.*, 2013*a*). However, no direct evidence that the regulation of a Ca^2+^-ATPase plays a direct role in shaping stimulus-induced Ca^2+^ signatures has been reported in complex organisms *in vivo*.

Using the *A. thaliana* PM-localized Ca^2+^-ATPase isoform ACA8, we have investigated if and how a Ca^2+^ pump and its fine tuning can affect the shape of a Ca^2+^ signature. ACA8 is a IIB P-type ATPase that extrudes Ca^2+^ from the cytosol to the apoplast. ACA8 is widely expressed throughout the plant (Bonza *et al.*, 2000; Cerana *et al.*, 2006; Bonza and De Michelis, 2011). The conserved structure and the complex regulation of ACA8 are shared by other members of the type IIB Ca^2+^-ATPase subgroup (Brini, 2009; Bonza and Luoni, 2010; Bonza and De Michelis, 2011; Tidow *et al.*, 2012). ACA8 is characterized by an extended cytosolic N-terminal regulatory domain containing both an autoinhibitory region and two sites able to bind calmodulin (CaM) with different affinities: CaM binding suppresses autoinhibition, determining the activation of the pump (Bonza *et al.*, 2000; Luoni *et al.*, 2004, 2006; Bækgaard *et al*, 2006; Bonza and De Michelis, 2011; Tidow *et al.*, 2012). Besides CaM binding, acidic phospholipids are also able to counteract the autoinhibitory action of the N-terminus (Meneghelli *et al*, 2008). In addition, phosphorylation of several N-terminal serine residues surrounding both the high-affinity and low-affinity CaM-binding sites alters autoinhibition, CaM affinity, and/or the kinetics of interaction with CaM (Giacometti *et al.*, 2012). Altogether, this generates intricate regulatory mechanisms of the pump activity to which the reported alteration of both ACA8 mRNA and protein expression level adds a further degree of pump control governed by physiological stimuli such as developmental processes and stress perception (Romani *et al.*, 2004; Schiǿtt and Palmgren, 2005; Cerana *et al.*, 2006; Frei dit Frey *et al.*, 2012; Zhang *et al.*, 2014). For some N-terminal serine residues, evidence has been provided that phosphorylation is regulated by hormonal, environmental, or nutritional signals (Nühse *et al.*, 2003, 2004, 2007; Benschop *et al.*, 2007; Niittylä *et al.*, 2007; Chen *et al.*, 2010). However, the identity of the protein kinases and phosphatases controlling the phosphorylation status of the ACA8 N-terminus remains to be determined. *In vitro* phosphorylation assays with calcium-dependent protein kinases (CDPKs) indicated that the N-terminus can be phosphorylated by this class of protein kinases on Ser19 and Ser22 (Giacometti *et al.*, 2012). On the other hand, the families of protein kinases involved in the *in vivo* phosphorylation of ACA8 have remained unknown so far. One subgroup of the plant SnRK (Sucrose non-fermenting Related Kinase; Hrabak *et al.*, 2003; Nühse *et al.*, 2004) family is directly involved in Ca^2+^ sensing: these kinases are designated CBL-interacting protein kinases (CIPKs; Luan, 2009; Batistič and Kudla, 2009; Weinl and Kudla, 2009) for their ability to interact with the calcineurin-B like Ca^2+^ sensor proteins (CBLs; Hrabak *et al.*, 2003). In *A. thaliana*, the 26 members of CIPKs are cytoplasmic autoinhibited kinases that interact differently with 10 CBLs, which target them to specific membranes and/or modulate their activity (Kolukisaoglu *et al.*, 2004; Luan, 2009; Batistič *et al.*, 2010; Kudla *et al.*, 2010; Hashimoto and Kudla, 2011).

In this work, we identify by two-hybrid screening, two isoforms of CIPKs as interactors of the ACA8 N-terminal region and by bimolecular fluorescence complementation (BiFC) assay experiments that the identified CIPKs interact with full-length ACA8 *in planta* at the PM and that both kinases can phosphorylate the ACA8 N-terminal region *in vitro*. Moreover, we demonstrate that ACA8 is involved in the response to wounding-related signals in Arabidopsis leaves and roots and that its phosphoregulation by the CBL1–CIPK9 complexes shapes *in vivo* the cytosolic Ca^2+^ signature induced by mechanical wounding of *Nicotiana benthamiana* leaves.

## Materials and methods

### Plasmid constructs

For yeast two-hybrid screening, the coding sequence (CDS) of the first 116 amino acids [Met1–1le16 (^1^M–I^116^)] of ACA8 inserted in the *Escherichia coli* expression vector pET15b (Luoni *et al.*, 2004) was subcloned into the activation domain (AD) vector pGADT7 (Clontech, USA; kindly provided by L. Colombo, Department of Biosciences, University of Milan, Italy), taking advantage of the restriction sites *Nde*I and *Bam*HI already present at the 5' and 3' ends, respectively, of the CDS of ACA8.

For the fusion at the C-terminal end of ACA8 with green fluorescent protein (GFP), the complete CDS of ACA8 was amplified by PCR with Phusion^®^ High-Fidelity DNA Polymerase (New England Biolabs, USA) according to the manufacturer’s protocol. Wild-type (wt) full-length (FL) ACA8 inserted in the YES2 vector (Bonza *et al.*, 2004) was used as template and the entire CDS was amplified with the following specific oligonucleotides: (S) 5' TCCG*C*TGGAGATGACGAGTCTCTTGAAGTCATCG and (AS) 5' CATATCCCGGGGAGTGAACCTTCTCCAGACGA. An *Xho*I and an *Xma*I restriction site (underlined) were added at the 5' and 3' ends, respectively, during amplification. The AS primer also eliminates the stop codon at the 3' end of the ACA8 FL CDS. Following digestion by *Xho*I/*Xma*I, the ACA8 FL CDS was inserted into the plant expression vector pGPTVII (Waadt *et al.*, 2008) under the control of the *Cauliflower mosaic virus* (CaMV) 35S promoter at the 5' end of the CDS of ACA8 (ACA8::GFP). Absence of errors was confirmed by sequencing.

The ACA8 FL CDS was also inserted into the pSPYCE(M)_155 vector (Waadt *et al.*, 2008) under the control of the CaMV 35S promoter for the fusion with the last 155 amino acids of the yellow fluorescent protein (YFP) C-terminus (YC). This pSPYCE(M)::ACA8 vector was used for BiFC studies in combinations with partners pSPYNE(R)::CIPK9, pSPYNE(R)::CIPK14, and pSPYNE(R)::CIPK7. BiFC assay has also been performed combining pSPYNE(R)::CIPK9, pSPYNE(R)::CIPK14, or pSPYNE(R)::CIPK7 with pSPYCE(M)::CBL1 (Batistič *et al.*, 2008, 2010).

The Cameleon targeted to the nucleus (NUP::YC3.6) was constructed including the *Xenopus laevis* nucleoplasmin (NUP) CDS (Badminton *et al.*, 1995; van der Luit *et al.*, 1999) under the control of the CaMV 35S promoter, placed in-frame with the CDS of the Yellow Cameleon 3.6 fluorescence resonance energy transfer (FRET)-based Ca^2+^ sensor (Cameleon YC3.6; Nagai *et al.*, 2004). The whole chimeric gene (NUP::YC3.6) was inserted into the *Kpn*I site of the *Agrobacterium tumefaciens* binary vector pGreen (0179) (Hellens *et al.*, 2000).

### Yeast two-hybrid assays

Construct combinations of the ACA8 N-terminus in the AD vector pGADT7 with the CDS of all 26 isoforms of CIPKs cloned into the BD vector pGBT9.BS (Kolukisaoglu *et al.*, 2004) were transformed into the yeast strain PJ69-4A (James *et al.*, 1996) using a lithium acetate/polyethylene glycol method (Waadt *et al.*, 2008) and selected on SD agar medium lacking Trp and Leu (SD-W-L) composed as followed: 900 mg l^–1^ HSM drop-out –His –Leu –Trp –Ura (FOREMEDIUM LTD, Hunstanton, UK), 200 mg l^–1^ uracil (Fluka Chemie GmbH, Germany), 200 mg l^–1^ His (Sigma-Aldrich, Stenheim, Germany), 2% glucose (FOREMEDIUM LTD), and 6.9 g l^–1^ yeast nitrogen base (FOREMEDIUM LTD) in ddH_2_O. To verify the interaction of the two proteins, all the positive transformants were grown on SD-W-L liquid medium at 30 °C for 16 h and 10-fold serial dilutions (10^–1^ to 10^–4^) of the transformants were prepared starting from an *A*_600_=1. A 5 μl drop for each dilution was spotted on agar plates of SD medium lacking Trp, Leu, and His (SD-W-L-H) and supplemented with 2.5 mM 3-amino-1,2,4-triazole (3AT; Sigma-Aldrich, Stenheim, Germany). Plates were incubated at 30 °C for 3–5 d.

### Transient expression in Nicotiana benthamiana leaves and wounding procedure

Plants were cultivated for 5–6 weeks in a greenhouse under a 12 h light/12 h dark cycle with 60% atmospheric humidity and at 22/18 °C. Leaf infiltration was performed using the *A. tumefaciens* GV3101/pMP90 strain carrying the specified constructs (see the Results for details) together with the p19-enhanced expression system (Voinnet *et al.*, 2003) and according to the method described by Waadt and Kudla (2008). After infiltration, plants were kept for incubation (3–5 d) under conditions as described above.

Small pieces (~1 cm×1 cm) from agroinfiltrated tobacco leaves were cut and gently placed on a homemade open top chamber (lower epidermis facing the coverslip, and upper epidermis directly in air) and wounding was then performed by gently pressing the lamina with laboratory forceps (Supplementary Video S1 at *JXB* online).

### Arabidopsis thaliana plant material

Arabidopsis seedlings used in this study were of the Columbia ecotype (Col-0). Homozygous T-DNA lines for ACA8 (GK-688H09) were a generous gift of Silke Robatzek (The Sainsbury Laboratory, Norwich, UK). The *Agrobacteriumtumefaciens* strain carrying the cytosol-localized Cameleon probe NES::YC3.6 (Nagai *et al.*, 2004; Krebs *et al.*, 2012) was used to transform the *aca8* knock-out T-DNA line of Arabidopsis by the floral-dip method (Clough and Bent, 1998). Four Arabidopsis independent transgenic lines were selected and verified for homozygous T-DNA insertion in *ACA8* by PCR using the same primers reported in Frei dit Frey *et al.* (2012). Two independent lines were employed for imaging experiments. Experiments were carried out on seedlings and mature plants of T_2_ generations.

### Arabidopsis seedling preparation for Ca^2+^ imaging, ATP treatment, and leaf wounding

Seeds of Arabidopsis were surface sterilized by vapour-phase sterilization (Clough and Bent, 1998) and plated on MS/2 medium [Murashige and Skoog (MS) medium including vitamins; Duchefa, The Netherlands] (Murashige and Skoog, 1962) supplemented with 0.1% (w/v) sucrose, 0.05% (w/v) MES, pH 5.8 adjusted with KOH, and solidified with 0.8% (w/v) plant agar (Duchefa). After stratification at 4 °C in the dark for 2–3 d, seeds were transferred to the growth chamber with 16/8 h cycles of light (70 μmol m^−2^ s^−1^) at 24 °C.

For root cell imaging, 7-day-old seedlings grown vertically were prepared according to Behera and Kudla (2013) in dedicated chambers and overlaid with wet cotton in order to perfuse the root continuously with the imaging solution (5 mM KCl, 10 mM MES, 10 mM Ca^2+^ pH 5.8 adjusted with Tris). The shoot was not submerged in the solution. External ATP was added at the reported concentrations as sodium salt to the chamber by perfusion in the same imaging solution. In order to prevent an imaging solution acidification occurring when higher ATP concentrations were used, the ATP sodium salt was prepared as a 200 mM stock buffered at pH 7.4 with NaOH.

Leaves of 4-week-old Arabidopsis plants were used to carry out wounding experiments. Each leaf was detached just before the experiment and treated as reported for the wounding experiments carried out in *N. benthamiana*.

### Confocal microscopy analysis and time-lapse Ca^2+^ imaging

Confocal microscopy analysis for the ACA8::GFP localization study and BiFC assays was performed as previously described in detail in Batistič *et al.* (2008, 2010). Before wounding, we checked leaves for the presence of the fluorescence emitted from the different expressed fluorescent proteins [GFP, YFP, cpVenus/Cameleon, and orange fluorescent protein (OFP)] in order to confirm their efficient co-expression. For the detection of GFP, YFP, and cpVenus fluorescence, we excited the agroinfiltrated leaves with the argon laser at 488 nm and the emission was collected between 505 nm and 540 nm. For OFP detection, we excited the leaves at 561 nm with an He/Ne laser and the emission was collected between 575 nm and 625 nm. Only leaves in which simultaneous co-expression of all partners was confirmed were subjected to wounding and used for Ca^2+^ imaging analysis. All these analyses were performed using an inverted microscope, Leica DMIRE2, equipped with a Leica TCS SP2 laser scanning device (Leica, Germany).

For Ca^2+^ imaging analysis, we used an inverted fluorescence Nikon microscope (Ti-E; http://www.nikon.com/) with a CFI ×4 NA (numerical aperture) 0.13 dry objective for the tobacco leaves or a ×20 NA 0.75 for seedling root. Excitation light was produced by a fluorescent lamp (Prior Lumen 200 PRO; Prior Scientific; http://www.prior.com) at 440 nm (436/20 nm) set to 20% (for Arabidopsis roots) and 50% (for tobacco and Arabidopsis leaves). Images were collected with a Hamamatsu Dual CCD camera (ORCA-D2; http://www.hamamatsu.com/). For Cameleon analysis, the FRET cyan fluorescent protein (CFP)/YFP optical block A11400-03 (emission 1, 483/32 nm for CFP; emission 2, 542/27 nm for FRET) with a dichroic 510 nm mirror (Hamamatsu) was used for the simultaneous CFP and cpVenus acquisitions. Exposure time was 400 ms with a 4 × 4 CCD binning for nuclear and cytosolic Cameleon in tobacco leaf cells as well as for cytosolic Cameleon in Arabidopsis leaf cells. An exposure time of 200 ms was chosen for Arabidopsis roots with a 2 × 2 CCD binning. Images were acquired every 2 s for leaves and 5 s for Arabidopsis roots. Filters and the dichroic mirror were purchased from Chroma Technology (http://www.chroma.com/). NIS-Elements (Nikon; http://www.nis-elements.com/) was used as a platform to control microscope, illuminator, camera, and post-acquisition analyses. For Cameleon analysis in tobacco leaves, cpVenus and CFP emissions of the analysed regions of interest (ROIs), corresponding to single nuclei surrounding the wounded region, were used for the ratio (R) calculation (FRET cpVenus/CFP), normalized to the initial ratio (R_0_) and plotted versus time (ΔR/R_0_). In the case of Arabidopsis imaging, single fluorescences of cpVenus and CFP corresponding to the entire meristematic zone of the root tip (Bonza *et al.*, 2013*a*) or of the leaf area surrounding the wounding site (~1 mm^2^) were background subtracted before calculation of the ratio. Background correction allowed us to compare directly both the normalized and the raw ratios of the two genotypes (wt and *aca8*).

Wounding of tobacco leaves, performed by gently pressing the lamina with forceps, can determine a focus shift which modifies the ΔR/R_0_ value, so that the final baseline is different from the initial one: only cells in which this difference was lower than 20% of the height of the first peak, and the first peak was neat and sharp were used for analysis; at least 30 cells of 8–13 independent leaves of at least four different plants from 3–7 independent co-infiltration experiments were analysed for each condition.

### Expression and purification of the 6His-tagged ACA8 N-terminus

The vector coding for the 6His-tagged ACA8 N-terminus (6His-^1^M–I^116^), produced as described in Luoni *et al.* (2004) was used to transform *Escherichia coli* strain BL21(DE3)-pLysE (Merck KGaA, Germany) by standard procedures. Purification of the fusion protein was performed as described (Luoni *et al.*, 2004).

### Wheat germ-based cell-free CIPK synthesis and protein purification

StrepII-tagged CIPK9 and CIPK14 proteins were synthesized using an RTS 500 wheat germ CECF kit (Thermo Fisher, USA) following the manufacturer’s instructions. For affinity purification, each *in vitro* translation reaction (1 ml) was mixed with 0.8 ml of Strep-Tactin Macroprep (IBA, GmbH, Germany) and incubated for 30 min at 4 °C. StrepII-tagged proteins were eluted by gravity flow in elution buffer (100 mM Tris pH 8.0, 150 mM NaCl, and 2.5 mM desthiobiotin) and collected in 0.4 ml fractions. The abundance of the purified proteins was confirmed by western blot analysis using Strep-Tactin–horseradish peroxidase (HRP) conjugate (1:5000; IBA). In addition, SDS–PAGE followed by Coomassie staining (R-250) was performed to estimate the concentration of each purified protein. Stained bands of the purified proteins were compared with known amounts of BSA standard. The concentration of StrepII–CIPK9 and StrepII–CIPK14 purified fraction was between 20 ng μl^–1^ and 30 ng μl^–1^.

### In vitro *phosphorylation assays*

For the kinase assay with CIPKs, purified glutathione *S*-transferase (GST) or ACA8 6His^1^M–I^116^ (700 ng per sample) and StrepII-tagged CIPK9 or CIPK14 proteins (60 ng per sample) were incubated for 30 min at 30 °C in 24 μl reactions that contained 66.7 mM Tris pH 8.0, 100 mM NaCl, 5 mM MnSO_4_, 0.5 mM CaCl_2_, 2 mM DTT, 10 μM ATP, and 4 μCi of [γ-^32^P]ATP. Reactions were stopped by addition of 20 mM EDTA, solubilized by addition of Laemmli buffer (Laemmli, 1970), and then subjected to SDS–PAGE on a 16% polyacrylamide gel. SDS gels were fixed by Coomassie staining, and radioactively labelled proteins were visualized by autoradiography. All experiments were repeated at least three times with similar results.

## Results and Discussion

### ACA8 interacts with CIPK9 and CIPK14

**Fig. 1.**
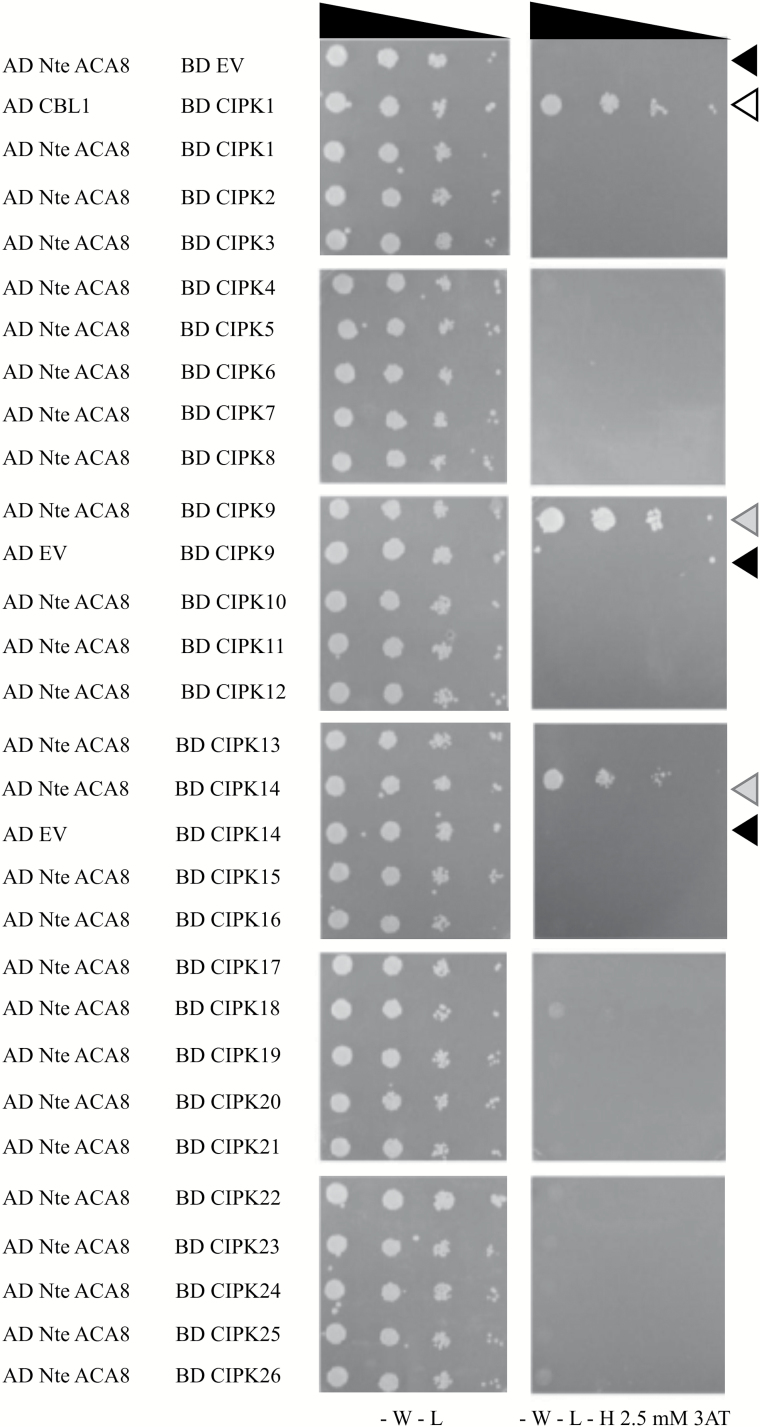
CIPK9 and CIPK14 interact with the ACA8 N-terminus in yeast two-hybrid assay. Serial dilutions of yeast PJ69-4A transformed with the ACA8 N-terminus (N-te) in the AD vector and all 26 CIPKs in BD vectors. Positive control: transformation with AD CBL1/BD CIPK1 vectors (white arrow; D’Angelo *et al.*, 2006). Negative controls: transformation with AD ACA8 N-te/BD EV, BD CIPK9/AD EV, or BD CIPK14/AD EV (black arrows). Growth on selective SD-W-L-H medium supplemented with 2.5 mM 3AT indicates the interaction of ACA8 N-te with CIPK9 and CIPK14 (grey arrows). EV, empty vector; AD, activation domain; BD, binding domain.

In the ACA8 N-terminal regulatory portion, several serine residues that are phosphorylated *in vivo* were previously identified. Their phosphorylation state is enhanced by treatment with abscisic acid and gibberellins (Ser27 and Ser29; Chen *et al.*, 2010) or with the elicitor flagellin (Ser27 and Ser99; Nühse *et al.*, 2003, 2004, 2007; Benschop *et al.*, 2007), or down-regulated in response to sucrose administration to cultured cells (Ser22; Niittylä *et al.*, 2007). Biochemical analysis of ACA8 S/D mutants clearly demonstrated that phosphorylation of serine residues can affect ACA8 activity both by hampering the autoinhibitory action of the N-terminus and by changing the kinetics of activation by CaM binding and de-activation by CaM release, and, in some instances, ACA8 affinity for CaM (Giacometti *et al.*, 2012). We therefore sought to identify the kinase(s) responsible for ACA8 phosphorylation and regulation. To this end, we performed a yeast two-hybrid screening using the ACA8 N-terminus as the bait and all 26 CIPK isoforms of Arabidopsis as preys. On the selective medium, only yeast cells co-transformed with the ACA8 N-terminus in combination with two of the 26 kinases, namely CIPK9 or CIPK14, grew as the positive control (Fig. 1), strongly suggesting an interaction of these two isoforms with the ACA8 N-terminus. Combinations of both CIPK9 and CIPK14 with the empty vector used as negative control clearly showed no growth (Fig. 1), thus excluding the possibility that the growth of the two co-transformants could depend on transactivation due to the presence of the kinases themselves.

**Fig. 2.**
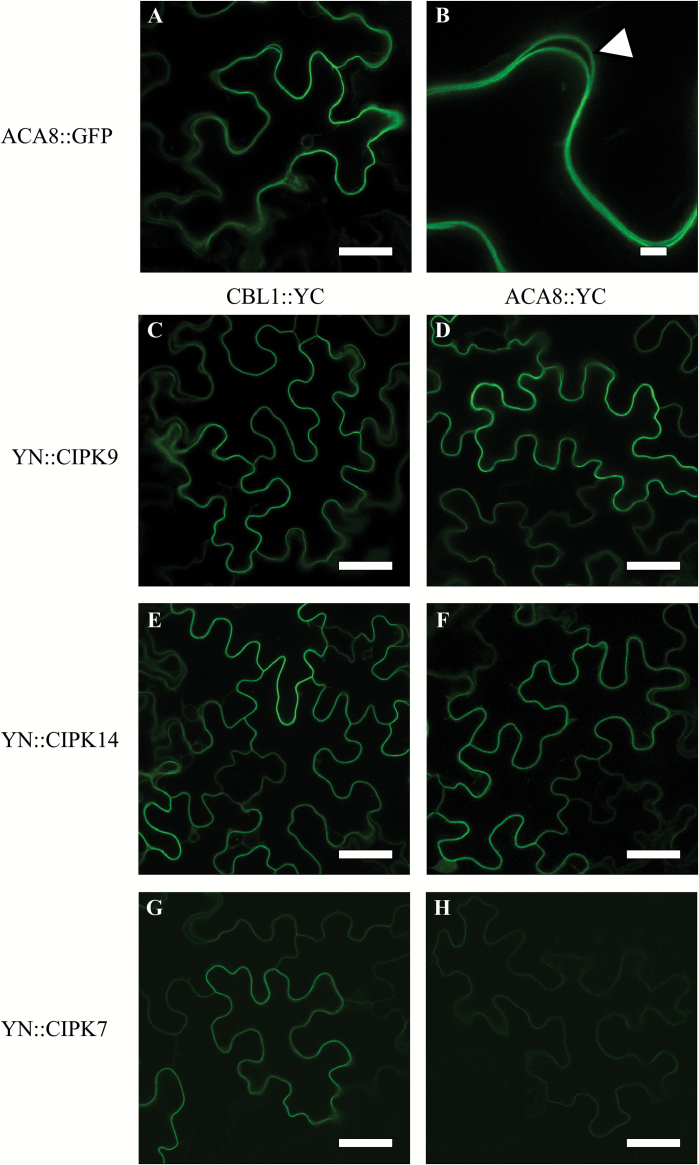
CIPK9 and CIPK14 interact with CBL1 and ACA8 in BiFC assay. (A and B) GFP fluorescence associated with ACA8 in *N. benthamiana* leaves marks the cell periphery. The focus image (B) is a 5-fold magnification of the image in (A). The white arrow in the magnified image indicates the pattern of signal distribution in two neighbouring cells. (C–H) Biomolecular fluorescence complementation (BiFC) analyses of YN::CIPK9, YN::CIPK14, and YN::CIPK7 with, respectively, CBL1::YC (C, E, G) and ACA8::YC (D, F, H) in transiently transformed *N. benthamiana* leaves. CIPK9, CIPK14, and, to a lesser extent, CIPK7 show interaction with ACA8 as indicated by the reconstitution of the YFP fluorescence (reported as green colour) at the cell periphery that represents the plasma membrane. Results, collected 4 d after infiltration with *A. tumefaciens*, are from one experiment representative of at least five independent experiments. Scale bars=40 μm.

In order to test if CIPK9 and CIPK14 also interact with the FL version of ACA8, we followed an *in planta* approach. As first we verified that ACA8 FL protein fused to GFP correctly localized at the PM of plant cells, by analysing the subcellular distribution of ACA8–GFP fusion protein in *N. benthamiana* leaves after transient transformation with *A. tumefaciens*. This experiment revealed that the fluorescent signal was uniformly distributed at the cell perimeter as expected for a PM localization (Fig. 2A, B). We then performed a series of BiFC experiments, with different combinations of CIPKs, CBLs, and ACA8 to allow a direct visualization of protein–protein interactions *in vivo* (Waadt and Kudla, 2008; Kudla and Bock, 2016).

As a rule, CIPKs are cytoplasmic proteins and their final cellular localization is promoted by interaction with other partners, primarily CBLs (Waadt *et al.*, 2008; Batistič *et al.*, 2010). It has been reported that CBL2 and CBL3 mediate targeting of CIPK14 and CIPK9 to the tonoplast (Batistič *et al.*, 2010; Tang *et al.*, 2015) and CIPK14 localizes at the PM upon interaction with CBL8 (Batistič *et al.*, 2010). Thus, we performed a series of BiFC experiments in which we used CBL1::YC, a well-characterized CBL exclusively localized at the PM (Batistič *et al.*, 2010), in combination with YN::CIPK9, YN::CIPK14, or YN::CIPK7. The reconstituted YFP signal (Fig. 2D, F, H) was evident and again was confined at the perimeter of the cells, showing that the three kinases are able to interact with CBL1 which targets them to the same membrane compartment as ACA8. Clear evidence shows that channels can also recruit the CIPKs to membrane locations independently of the presence of added CBLs (e.g. AKT1 with CIPK23 and AKT2 with CIPK6) (Xu *et al.*, 2006; Held *et al.*, 2011). Hence, independently of the presence of CBLs, we used the ACA8 FL protein fused to the C-terminal part of YFP (ACA8::YC) together with CIPK9, CIPK14, or CIPK7 fused to the N-terminal part of YFP (YN::CIPK9, YN::CIPK14, and YN::CIPK7). The expression of both ACA8–CIPK9 and ACA8–CIPK14 combinations revealed a strong YFP fluorescence signal at the periphery of the cells (Fig. 2C, E), indicating the YFP reconstitution due to the close proximity of ACA8 and the kinases at the PM, thus supporting the results obtained by yeast two-hybrid screening using the sole ACA8 N-terminus. Despite CIPK7 not interacting with the ACA8 N-terminus in the two-hybrid assay (Fig. 1), when *N. benthamiana* leaves were transformed with ACA8 in combination with this CIPK isoform a YFP fluorescent signal was still detectable, although of much lower intensity compared with the other two tested combinations (Fig. 2G). This latter observation does not exclude that CIPK7 *in planta* also represents a putative ACA8 partner; however, that it might be an artefact, caused by BiFC stabilization, resulting in a false-positive fluorescence signal (Kudla and Bock, 2016), cannot be excluded. However, overall, these results confirm that *in planta* the CIPK members (including those identified in the two-hybrid experiment) are able to interact with ACA8, supporting the evidence that targeting of CIPKs depends on their interaction not only with CBLs, but also with their putative targets (e.g. channels or transporters).

### CIPK9 and CIPK14 phosphorylate ACA8 in vitro

**Fig. 3.**
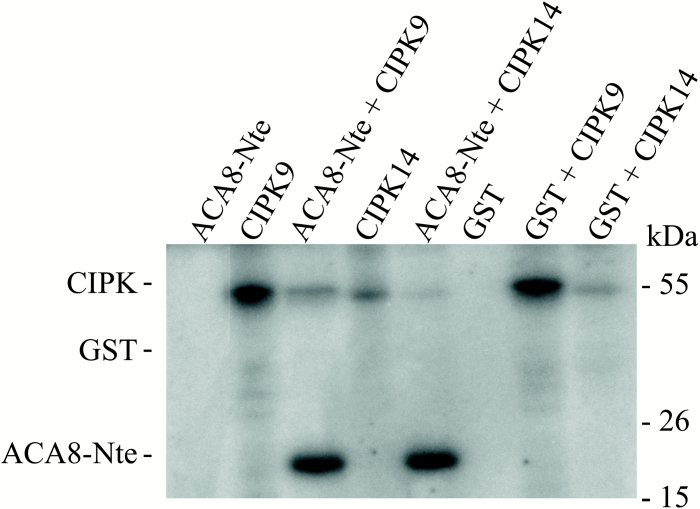
CIPK9 and CIPK14 specifically phosphorylate the ACA8 N-terminus *in vitro*. *In vitro* phosphorylation of the N-terminus (N-te) of ACA8 using StrepII-tagged recombinant CIPK9 or StrepII-tagged recombinant CIPK14. As negative controls, phosphorylation samples using purified GST as substrate or with N-te or GST alone were used. Assays were performed as described in the Materials and methods. Samples were solubilized and aliquots corresponding to 700 ng of N-te or GST were subjected to SDS–PAGE and autoradiography. Results are from one experiment representative of three. Numbers on the right refer to the mass of molecular weight markers.

Having demonstrated that ACA8 was able to interact primarily with both CIPK9 and CIPK14, we investigated whether these two isoforms were able to phosphorylate the N-terminus of ACA8. Recombinant StrepII-tagged CIPK9 and CIPK14 were synthesized using the wheat germ *in vitro* transcription/translation system, already described as a successful method to produce suitable amount of active CIPKs (Hashimoto *et al.*, 2012), while the His-tagged peptide corresponding to the ^1^M–I^116^ N-terminal sequence of ACA8 was expressed in *E. coli* (Luoni *et al.*, 2004). Proteins were purified by affinity chromatography as described in the Materials and methods. Figure 3 shows that both kinases (CIPK9 and CIPK14) efficiently phosphorylated the His-tagged ACA8 N-terminus. Conversely, no signal corresponding to GST phosphorylation was detectable, indicating that the ACA8 N-terminus is a specific substrate for CIPK9 and CIPK14. As expected, both kinases displayed autophosphorylation (Hashimoto *et al.*, 2012). In the presence of the ACA8 N-terminus, signals corresponding to kinase autophosphorylation were drastically reduced. This reduction in detectable autophosphorylation probably depends on the experimental conditions, in which ACA8 N-terminus was in high molar excess (~50-fold) compared with the kinase. This further confirms that the ACA8 N-terminus is a substrate for both CIPK9 and CIPK14. Furthermore, these results support the conclusion drawn from the two-hybrid screening that a functional interaction between the two CIPKs and ACA8 occurs at the N-terminus of the ATPase. Ser27 in the N-terminus of ACA8 is a putative substrate of SnRKs: phosphorylation by CIPK9 is diminished in the S27A mutant of the ACA8 N-terminus but not abolished, indicating that phosphorylation, at least under the applied conditions, occurs at multiple sites which could not be unequivocally resolved (data not shown).

### ACA8 interaction with CBL1–CIPK9 complexes influences stimulus-induced Ca^2+^ dynamics in vivo

We hypothesized that CIPK-dependent ACA8 phosphorylation could represent a mechanism for fine-tuning of Ca^2+^ efflux in response to different signals, that is likely to play a role in determining the magnitude and duration of the stimulus-induced Ca^2+^ spike. Hence, to unravel the putative effects of ACA8 phosphorylation by CIPKs on Ca^2+^ dynamics, we investigated the effect of the simultaneous overexpression of ACA8, CIPK9, and CBL1 on the regulation of cellular Ca^2+^ dynamics. To this end, we transiently co-expressed fluorescence labelled versions of the proteins in the same *N. benthamiana* leaf cells. In order to analyse the Ca^2+^ dynamics *in vivo*, we decided to employ the ratiometric FRET-based probe yellow Cameleon YC3.6 (Nagai *et al.*, 2004) that successfully allows the study of Ca^2+^ dynamics in specific plant organs and single cells (Costa and Kudla, 2015). A cytosol-localized Cameleon NES::YC3.6 is available (Krebs *et al.*, 2012). However, the large central vacuole of tobacco epidermal cells would bring the cytosol and the PM in close contact, consequently allowing for fluorescence interference between the emissions of the cytosol-localized Cameleon probe and of the fluorescent overexpressed proteins. This effect would cause a drastic reduction in the dynamic range of the probe. To minimize this problem, we took advantage of our direct experimental observations that in plants nuclear Ca^2+^ transients, triggered by several stimuli, can often mirror those generated in the cytosol (Loro *et al.*, 2012). Hence, we generated a nuclear-localized version of the YC3.6 under the control of a constitutive promoter (CaMV 35S). To target the YC3.6 to the nucleus and reduce mistargeting, we fused at its N-terminal end an *X. laevis* nucleoplasmin, a nuclear protein previously used to target the aequorin Ca^2+^ reporter efficiently to tobacco nuclei (van der Luit *et al.*, 1999). The obtained construct was termed NUP::YC3.6, and confocal analyses confirmed its proper nuclear localization in *N. benthamiana* leaf cells (Supplementary Fig. S1).

**Fig. 4.**
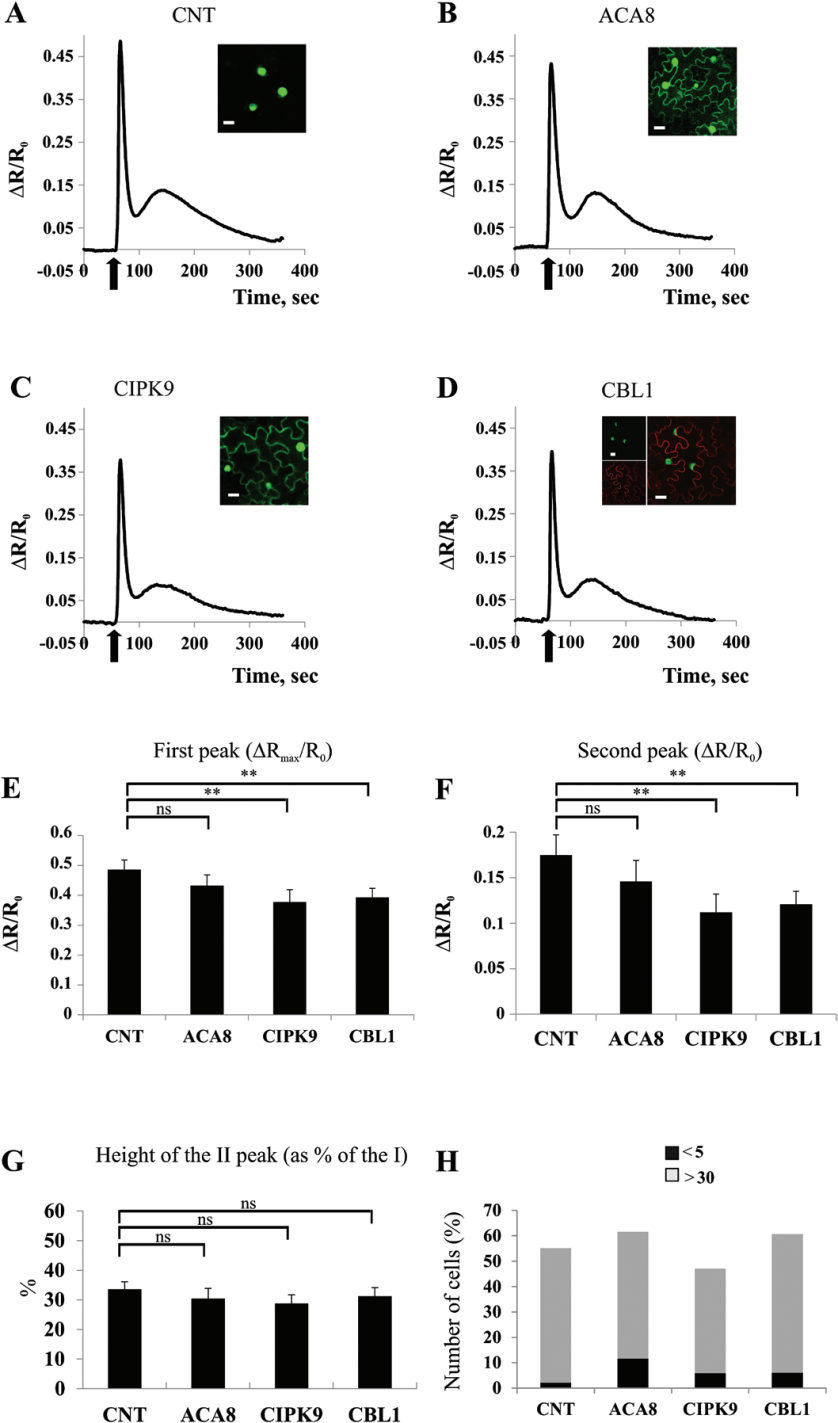
Ca^2+^ signatures induced by leaf mechanical wounding in tobacco leaves expressing each of the tested proteins alone. (A–D) Nuclear Ca^2+^ concentration monitoring in *N. benthamiana* leaf cells transiently expressing NUP::YC3.6 alone (CNT, A) or co-expressing NUP::YC3.6 and ACA8::GFP (B), NUP::YC3.6 and CIPK9::GFP (C), or NUP::YC3.6 and CBL1::OFP (D). Leaves were challenged with wounding (arrow), and FRET variations (normalized FRET cpVenus/CFP ratio reported as ΔR/R_0_) in single cells surrounding the wounded site were observed for ~400 s at 2 s intervals. Traces are the averages from the analysis of at least 25 independent cells. Insets: single plane confocal images of *N. benthamiana* epidermal cells from leaves used for wounding experiments, showing the simultaneous expression of the different expressed fluorescent proteins. (E and F) Comparison and statistical analysis of the height of the first and second peaks of the Ca^2+^ transients (as determined by single-cell analysis) reported as the normalized ΔR/R_0_ (±SEM), measured in the different tested conditions. (G) Mean height of the second peak expressed as a percentage of the height of the first peak (±SEM). Asterisks indicate statistically significant differences (**P<0.05, ns=not significant) calculated using Student’s *t*-test. (H) Comparison of the number of epidermal cell nuclei in which the height of the second peak is <5% or >30% of the height of the first one in *N. benthamiana* leaves infiltrated with the different combinations of *A. tumefaciens* harbouring the different plasmids.

**Fig. 5.**
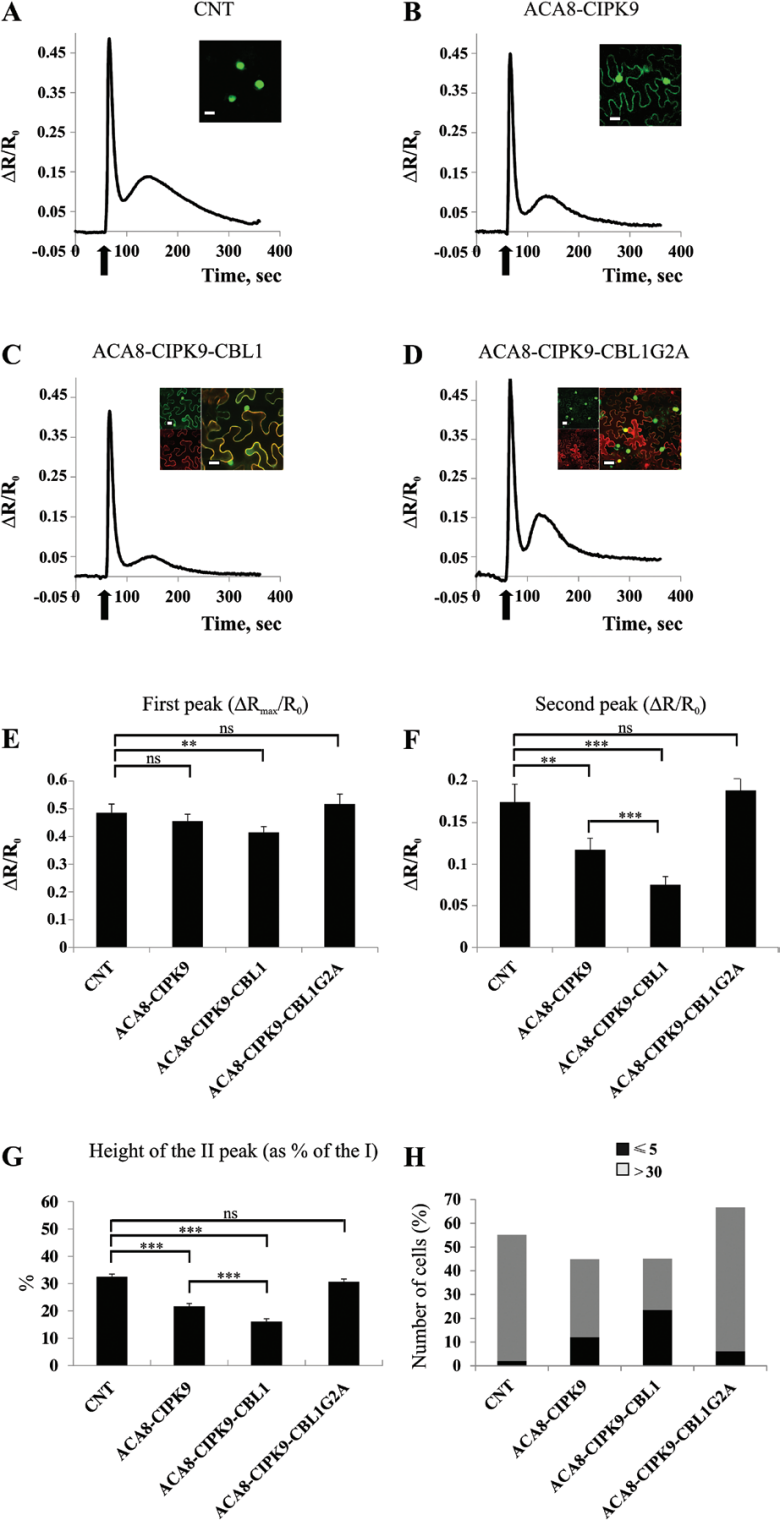
ACA8 interaction with CIPK9 and CBL1 influences Ca^2+^ signatures induced by leaf mechanical wounding. (A–D) Nuclear Ca^2+^ concentration monitoring in *N. benthamiana* leaf cells expressing NUP::YC3.6 alone (CNT, A) or co-expressing NUP::YC3.6, ACA8::YC and YN::CIPK9 (B), NUP::YC3.6, ACA8::YC, YN::CIPK9, and CBL1::OFP (C) and NUP::YC3.6, ACA8::YC, YN::CIPK9, and mutant CBL1-G2A::OFP (D). Leaves were challenged with wounding (arrow), and FRET variations (normalized FRET cpVenus/CFP ratio reported as ΔR/R_0_) in single cells surrounding the wounded site were observed for ~400 s at 2 s intervals. Traces are the averages from the analysis of at least 30 independent cells. Insets: single plane confocal images of *N. benthamiana* epidermal cells from co-infiltrated leaves used for wounding, showing the simultaneous expression of the different expressed fluorescent proteins. (E and F) Comparison and statistical analysis of the height of the first and second peaks of the Ca^2+^ transients (as determined by single-cell analysis) reported as the normalized ΔR/R_0_ (±SEM), measured in the different tested conditions. (G) Mean height of the second peak expressed as a percentage of the height of the first peak (±SEM). Asterisks indicate statistically significant differences (**P<0.05, ***P<0.01, ns=not significant) calculated using Student’s *t*-test. (H) Comparison of the number of epidermal cell nuclei in which the height of the second peak is <5% or >30% of the height of the first one in *N. benthamiana* leaves infiltrated with the different combinations of *A. tumefaciens* harbouring the different plasmids.

We decided to monitor cellular Ca^2+^ dynamics induced by wounding, a stimulus reported to induce reliable and reproducible cytosolic Ca^2+^ transients in Arabidopsis (Beneloujaephajri *et al.*, 2013; Supplementary Video S1). To confirm that nuclear Ca^2+^ dynamics also mirror cytosolic alterations in our experimental system, we first performed a series of experiments in which the wound-induced Ca^2+^ transients were compared in leaves of *N. benthamiana* plants transiently transformed with NES::YC3.6 (Krebs *et al.*, 2012) or NUP::YC3.6. The typical Ca^2+^ signature consisting of a first fast and steep increase of the FRET (indicating an increase in Ca^2+^ concentration) followed by a second slower, smaller, and more sustained peak (Beneloujaephajri *et al.*, 2013) was observed (Figs 4A, 5A; Supplementary Fig. S2). The amplitude of the second peak was slightly higher in the nucleus than in the cytosol (Supplementary Fig. S2): this has already been reported for guard cells subjected to osmotic stress and might depend on the different behaviour of the probe in the two environments (Loro *et al.*, 2012). Nevertheless, with the resolution offered by our imaging analyses and sampling time of 2 s, we did not observe substantial differences between the kinetics of Ca^2+^ transients triggered by wounding in the two compartments, hence validating the use of NUP::YC3.6 as the cytosolic mirror, as previously done in both plant (Loro *et al.*, 2012) and mammalian cells (Giacomello *et al.*, 2010).

Accordingly, in further experiments, we monitored the FRET signal of NUP::YC3.6 to analyse the effect of overexpression of ACA8, CIPK9, and CBL1 (alone or in combination) on wounding-induced Ca^2+^ dynamics in *N. benthamiana* leaves. In every experiment, before challenging, we inspected the leaf by means of fluorescent and/or confocal microscopy (see the Materials and methods) for the presence of fluorescence originating from the differently expressed proteins in order to confirm their efficient co-expression (insets in Figs 4 and 5). The curves reported in Figs 4 and 5 represent the averages of the curves of >25 analysed independent cells proximal to the wounded site. Figure 4 shows that co-expression of NUP::YC3.6 together with each of the tested proteins on its own (ACA8::GFP, CIPK9::GFP, and CBL1::OFP; Batistič *et al.*, 2008; Liu *et al.*, 2013) resulted in a slight decrease of the intensity ratios compared with the control (CNT, corresponding to NUP::YC3.6 alone), which may be ascribed to interference by the highly expressed fluorescent proteins which reduces the dynamic range of the probe (Fig. 4A–D). Since the timing of the second peak was variable (between 1 min and 2 min after wounding), we performed single-cell analysis (Fig. 4E–H). Expression of CIPK9 or CBL1 significantly decreased the height of both the first and the second peak (Fig. 4E, F), but did not affect the ratio between the heights of the two peaks (Fig. 4G). Moreover, the number of cells in which the second peak was hardly detectable was very low in all the tested conditions, with ~50% of the cells showing a pronounced second peak (>30% of the first peak; Fig. 4H).

Co-expression of NUP::YC3.6 together with both ACA8 and CIPK9 (ACA8::YC-YN::CIPK9) or with the full set of proteins (ACA8::YC-YN::CIPK9 and CBL1::OFP) (Fig. 5) did not markedly affect the height of the first peak of the Ca^2+^ transient which was essentially the same in all conditions (Fig. 5A–C, E). The decline phase of the first peak was, however, somewhat quicker in cells expressing ACA8 and CIPK9 or the full set of proteins so that the (minimum) ratio measured 34 s after wounding was significantly (*P*<0.05) lower than that of the control (Fig. 5A–C). When NUP::YC3.6 was co-expressed together with both ACA8 and CIPK9 (ACA8::YC-YN::CIPK9), a significant reduction of the height of the second peak was observed (Fig. 5F). This effect was drastically accentuated when tobacco cells co-expressed ACA8 in association with CIPK9 and CBL1 (ACA8::YC-YN::CIPK9 CBL1::OFP) simultaneously (Fig. 5F). The alteration of the wounding-induced Ca^2+^ dynamic was also clearly evident when we normalized the height of the second Ca^2+^ peak to the height of the first one (Fig. 5G). Compared with CNT, *in vivo* co-expression of ACA8 with CIPK9, and more so with the additional presence of CBL1, raised the number of epidermal cells in which the height of second peak was hardly detectable (<5% of the height of the first one), while greatly decreasing the number of cells in which it was >30% of the first peak (Fig. 5H). These results overall confirm that, upon overexpression, ACA8 and CIPK9 interact independently of expression of CBL1 and indicate that this interaction affects ACA8 activity, thus provoking relevant functional consequences. Previous studies have demonstrated the ability of CIPKs to interact with and regulate several ion transporters and channels as well as other critical proteins involved in stimulus-specific accumulation of second messengers such as NADPH oxidase (Quintero *et al.*, 2002; Li *et al.*, 2006; Xu *et al.*, 2006; Held *et al.*, 2011; Drerup *et al.*, 2013; Li *et al.*, 2014). In all cases, only co-expression of the combination of both CBL and CIPK with the target was able to regulate it significantly, thus causing a substantial alteration of its activity in heterologous expression systems such as frog oocytes, yeast, or HEK cells (Quintero *et al.*, 2002; Li *et al.*, 2006; Xu *et al.*, 2006; Hashimoto *et al.*, 2012; Drerup *et al*, 2013; Li *et al.*, 2014). In our experimental conditions, co-expression of ACA8 with only CIPK9 was already sufficient to affect considerably the Ca^2+^ signature induced by mechanical wounding of *N. benthamiana* leaves. This effect might be ascribable to the stabilization of the ACA8–CIPK9 interaction due to BiFC and/or to the activity of tobacco CBLs. The reason why this effect is exacerbated upon co-expression of CBL1 could be dual: (i) by targeting a larger amount of CIPK9 to the PM, CBL1 would improve its interaction with ACA8; and/or (ii) it could stimulate CIPK9 kinase activity. In fact, it has been shown that CBLs are able to stimulate the activity of several isoforms of CIPK (Batistič *et al.*, 2010; Held *et al.*, 2011; Hashimoto *et al.*, 2012). To corroborate this conclusion further, we performed co-expression of ACA8 in association with CIPK9 and a mutated version of CBL1 (CBL1G2A::OFP). This mutation prevents CBL1 N-terminal myristoylation and consequently the correct targeting of CBL1 to the PM, resulting in cytoplasmic/nuclear localization of the protein (Batistič *et al.*, 2008). As can be seen in Fig. 5D, the red fluorescence associated with CBL1G2A::OFP was now diffused into the cell, confirming the cytoplasmic localization of the mutated Ca^2+^ sensor. Interestingly, co-expression of CBL1G2A with ACA8 and CIPK9 was not only ineffective in decreasing the height of the second peak of the Ca^2+^ transient evoked by mechanical wounding stimulation of *N. benthamiana* epidermal cells, but clearly reversed the effect of ACA8 and CIPK9 interaction: the height of the second peak was similar to that of Ca^2+^ traces measured in control cells expressing NUP::YC3.6 alone (Fig. 5D, F–H). We hypothesize that overexpression of CBL1G2A in the cytosol might sequester the majority of the CIPK9, preventing its correct localization at the PM and its interaction with ACA8. This is consistent with the faint BiFC signal at the PM ascribable to a few ACA8–CIPK9 interactions in these cells (Fig. 5D, inset). In agreement with this, overexpression of ACA8::GFP alone did not alter the shape of the Ca^2+^ signature that is just like that of the CNT (Fig. 4B) probably due to the autoinhibited status of the pump.

Altogether, these results establish that ACA8–CIPK9–CBL1 interaction *in vivo* accelerates the recovery phase of the first peak and greatly decreases the height of the second peak of the nuclear wounding-induced Ca^2+^ signature that mirrors that generated in the cytosol. Overall, the timing of these effects is congruent with the time necessary for the formation of the complex between ACA8, CIPK9, and CBL1, and for phosphoregulation of the pump to take place. Noticeably, S/D mutation of Ser27, which is localized in a consensus motif for SnRK and is at least one of the amino acid targets of phosphorylation by CIPK9 (data not shown), results in a partially deregulated ACA8 with faster kinetics of interaction with CaM (Giacometti *et al.*, 2012).

### Cytosolic Ca^2+^ signatures in response to both wounding and extracellular ATP (eATP) are altered in Arabidopsis aca8 loss-of-function mutants

**Fig. 6.**
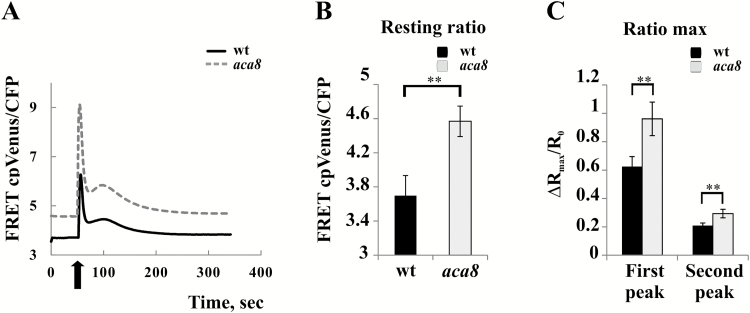
Cytosolic Ca^2+^ transients in response to wounding are altered in the *aca8* single knock-out mutant. Leaves of wt or *aca8* plants that constitutively express cytosolic YC3.6 were wounded (arrow). FRET changes were measured in a small area proximal to the wound site (~1 mm^2^). Traces (A) represent the raw FRET cp Venus/CFP ratio variations observed during the entire experiment and are the averages from the analysis of 23 independent wounding events performed on 23 independent leaves of 10 different plants for each genotype. (B) Statistical analysis of raw cpVenus/CFP ratios measured before challenging wt or *aca8* leaves with wounding. (C) Statistical analysis of the normalized cpVenus/CFP ratio of the first and second peaks of the Ca^2+^ transients measured in wt or *aca8* seedlings and reported as ΔR/R_0_. Values are means ±SEM. Asterisks indicate statistically significant differences (**P<0.05) calculated using Student’s *t*-test.

*In vivo* phosphorylation of ACA8 by CBL–CIPK complexes may increase pump activity, and thus the rate of Ca^2+^ extrusion through the PM, counteracting the effect of stimulus-induced Ca^2+^ influx and thus decreasing the height of the second peak of the wounding-induced Ca^2+^ transient. Although our data fully support this conclusion, the approach used might have the intrinsic bias of overexpressing ACA8 and its regulatory complex in a heterologous system, that already has its own set of PM-localized Ca^2+^ pumps, Ca^2+^ sensors, and kinases. To corroborate our results further by an independent approach, we thus decided to use an available *aca8* knock-out loss-of-function mutant of Arabidopsis (Frei dit Frey *et al.*, 2012). We thus evaluated the contribution of ACA8 to the shaping of the Ca^2+^ signature by monitoring cytosolic Ca^2+^ dynamics in leaves of 4-week-old *aca8* and wt Arabidopsis plants constitutively expressing the cytosol-localized NES::YC3.6 (Nagai *et al.*, 2004; Krebs *et al.*, 2012) challenged with wounding. Changes in FRET were analysed over ROIs which surrounded the wounding site that included several cells (~1 mm^2^ of leaf area). Both Ca^2+^ peaks, typical of the response, were significantly modified, being higher in *aca8* plants compared with the wt (Fig. 6A, C). This result clearly indicates that ACA8 is involved in the generation of the wounding-induced Ca^2+^ signature. Nevertheless, striking evidence was the different basal resting Ca^2+^ level observed in the two genotypes, with that of *aca8* significantly higher than that of the wt (Fig. 6A, B). Indeed, these different Ca^2+^ pre-stimulus levels might indicate that the altered Ca^2+^ dynamics observed in *aca8* may be dependent on adaptation to the lack of ACA8 in general rather than reflect a direct role of ACA8 in the response to wounding.

**Fig. 7.**
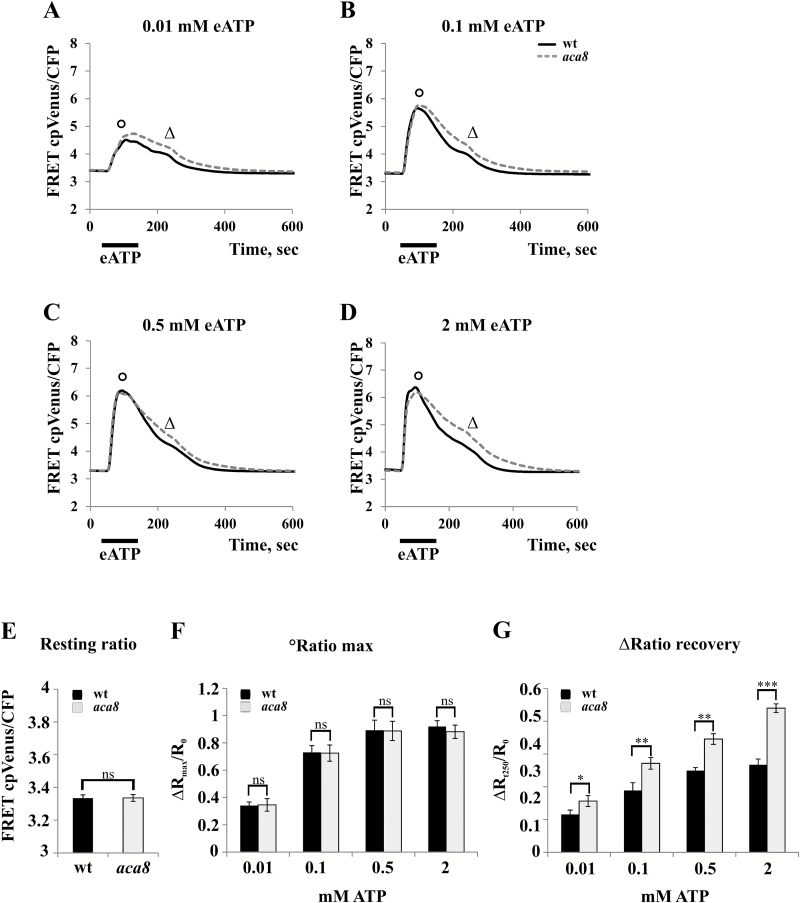
The cytosolic Ca^2+^ signature in response to extracellular ATP (eATP) is altered in the *aca8* single knock-out mutant. Root tips of 7-day-old wt or *aca8* seedlings expressing NES::YC3.6 were treated for the indicated time with the specified eATP concentrations. Traces (A–D) represent the raw FRET cp Venus/CFP ratio variations in root tips observed during the entire experiment. The analyses were performed by considering the entire imaged root. (E) Statistical analysis of resting raw ratios measured before challenging wt or *aca8* seedlings with eATP. (F) Statistical analysis of the normalized maximum FRET cpVenus/CFP ratio (°) reached after the administration of various eATP concentrations to wt or *aca8* seedlings and reported as ΔR_max_/R_0_. (G) Statistical analysis of the normalized cpVenus/CFP ratio measured in wt or *aca8* seedlings 250 s after challenging (Δ) and reported as ΔR/R_0_. Values are means ±SEM. Asterisks indicate statistically significant differences (*P<0.1, **P<0.05, ***P<0.01, ns=not significant) calculated using Student’s *t*-test.

Since experiments were carried out with mature plants, we tried also to analyse cytosolic Ca^2+^ changes in 7-day-old *aca8* and wt Arabidopsis seedlings. In young seedlings, potential adaptation effects could be limited, as also suggested by the absence of clear effects on the root length in *aca8* compared with the wt (data not shown). Besides, although largely co-expressed with the ACA10 isoform in the plant body, ACA8 represents the most expressed PM-localized ACA in the root tip region (Supplementary Fig S3; eFP Browser, http://bar.utoronto.ca/efp/cgi-bin/efpWeb.cgi;Winter *et al.*, 2007). Hence we chose this organ of young seedlings to investigate further the role of ACA8 in the regulation of Ca^2+^ transients. Since wounding of the small rootlets was not easily feasible under the microscope, as stimulus we used external ATP (eATP), that is strictly correlated to wounding, being released during physical damage, and known to induce defined cytosolic Ca^2+^ signatures in root tip cells (Tanaka *et al.*, 2010; Krebs *et al*, 2012; Loro *et al.*, 2012, 2013; Bonza *et al.*, 2013*a*). We treated wt and *aca8* Arabidopsis seedlings with increasing eATP concentrations [0.01–2 mM, a concentration range similar to that used in Choi *et al.* (2014) to mimic wound-induced effects] and analysed the FRET changes in the root tip (Fig. 7). In agreement with published data (Tanaka *et al.*, 2010; Loro *et al.*, 2012; Bonza *et al.*, 2013*a*), eATP administration resulted in a strong and sustained [Ca^2+^]_cyt_ increase in the entire subapical region of seedling roots, with kinetics that displayed a typical signature consisting of a first Ca^2+^ peak followed by a series of smaller sequential peaks. Due to the multicellular resolution of our analyses, the latter appeared as a continuous [Ca^2+^]_cyt_ decrease (Fig. 7; Costa *et al.*, 2013). An increase in eATP concentration led to a dose-dependent enhancement of the magnitude of the maximum FRET signal (Fig. 7A–D). Comparison of values corresponding to the raw resting ratio and the raw maximum ratio revealed that these parameters characterizing the Ca^2+^ signatures were identical in the wt and *aca8* (Fig. 7E, F), a result that was in contrast to what was observed in mature leaves. Hence, in seedlings, the existence of putative long-lasting effects due to the lack of ACA8 was missing, allowing us to define its role in the sole regulation of Ca^2+^ dynamics more easily. In fact, this new approach enabled us to detect clear differences in the kinetics of the decay phase of the curves which were evident even after administration of the lowest eATP concentration (Fig. 7A–D). As presented in the comparison of the raw ratios measured at 250 s from exposure (Fig. 7G), *aca8* exhibited a decelerated recovery (by ~30%) to basal [Ca^2+^]_cyt_ when compared with the wt. This alteration influenced the time required to reach basal [Ca^2+^]_cyt_ levels, with *aca8* that readjusted significantly later (328 ± 103 s and 553 ± 119 s for the wt and *aca8*, respectively, after 2 mM eATP treatment). This resulted in a more sustained [Ca^2+^]_cyt_ transient in mutant seedling roots compared with the wt.

In contrast to what was previously reported by using the biosensor AEQ (Frei dit Frey *et al.*, 2012), by means of our imaging-based method that allowed us to look at specific cell types with an increased spatial and temporal resolution, we were able to appreciate differences in the Ca^2+^ kinetics between the wt and the single mutant *aca8.*

Altogether, these results provide direct evidence that ACA8 plays a relevant role in the response to wounding (or to the wounding-related signal eATP) and, in agreement with the data obtained by transient expression of ACA8 and its regulatory circuit in *N. benthamiana* epidermal cells (Fig. 5), highlight that the impact of ACA8 activity on the kinetics of cytosolic Ca^2+^ signatures is mainly exerted in the recovery phase.

## Conclusions

Signal transduction mechanisms often depend on Ca^2+^-dependent phosphorylation events involved in activity modulation of target proteins to specifically transduce stimuli into accurate physiological responses (Boudsocq *et al.*, 2010; Dodd *et al.*, 2010; Kudla *et al.*, 2010; Hashimoto and Kudla, 2011, Steinhorst and Kudla, 2013).

Despite the functional overlap of PM-localized Ca^2+^-ATPase isoforms (Bonza and De Michelis, 2011; Bose *et al.*, 2011), our complementary analyses combining overexpression in *N. benthamiana* and investigation of an Arabidopsis loss-of-function mutant allowed us to identify ACA8 as a prominent pump involved in the termination of Ca^2+^ signals in response to wounding and eATP application. Moreover, in this work, we have identified a Ca^2+^-dependent regulatory circuit in which the PM Ca^2+^ pump ACA8 represents a target for CBL–CIPK complexes. This conclusion is based on our observations that (i) two isoforms of CIPK, namely CIPK9 and CIPK14, are able to interact with ACA8 *in vitro* and *in vivo* and phosphorylate ACA8 *in vitr*o; and (ii) ACA8 phosphoregulation by the CBL1–CIPK9 complex shapes the cytosolic Ca^2+^ transients induced by mechanical wounding of leaves *in vivo*.

Previous studies have demonstrated the ability of CBL–CIPK complexes to interact with and regulate several targets in heterologous expression systems such as frog oocytes, yeast, or HEK cells (Quintero *et al.*, 2002; Li *et al.*, 2006; Xu *et al.*, 2006; Held *et al.*, 2011; Drerup *et al.*, 2013; Li *et al.*, 2014). We have reconstituted the entire Ca^2+^-dependent CBL1–CIPK9–target regulatory circuit in tobacco. This allowed us to evaluate the function of a CBL–CIPK complexes *in planta* and to uncover how phosphorylation-mediated fine tuning of a Ca^2+^ pump crucially controls the appropriate formation of cytoplasmic Ca^2+^ signatures.

As a target of activation by CaM (Bonza *et al.*, 2000; Luoni *et al.*, 2004, 2006; Bækgaard *et al.*, 2006; Tidow *et al.*, 2012; Bonza *et al.*, 2013*b*), potential phosphoregulation by CDPK (Giacometti *et al.*, 2012), and phosphoregulation by the complex CBL–CIPKs, ACA8 is at the centre of an elaborate Ca^2+^-dependent regulatory network for the fine tuning of intracellular Ca^2+^ homeostasis. Fine tuning of spatial, temporal, and concentration parameters of Ca^2+^ signatures is probably fundamental for the ability of plants to elicit adequate responses to environmental cues that lead to growth adaptation.

## Supplementary data

Supplementary data are available at *JXB* online.

Fig. S1. Subcellular localization of the NUP::YC3.6 probe in a representative tobacco epidermal leaf cell.

Fig. S2. Comparison of wounding-induced Ca^2+^ transients in leaves of *N. benthamiana* plants transiently transformed with NES::YC3.6 or NUP::YC3.6.

Fig. S3. Expression levels of PM-localized ACA isoforms in roots of *Arabidopsis thaliana.*

Video S1. Series of nuclear Ca^2+^ ratio images of a *N. benthamiana* leaf subjected to wounding

## Supplementary Material

supplementary_Figures_S1_S3

supplementary_video_S1
