# Supplementary material for: Ca2+-dependent phosphoregulation of the plasma membrane Ca2+-ATPase ACA8 modulates stimulus-induced calcium signatures
Source: J Exp Bot. 2017 May 20;68(12):3215–30. doi: 10.1093/jxb/erx162 (PMC5853299; doi:10.1093/jxb/erx162)
Supplement: supplementary_Figures_S1_S3 [file erx162_suppl_supplementary_figures_s1_s3.pdf]

Ca2+-dependent phosporegulation of the plasma membrane Ca2+ATPase ACA8 modulates stimulus-induced Ca2+ signatures

Alex Costa , Laura Luoni, Claudia A Marrano, Kenji Hashimoto, Philipp Köster, Sonia Giacometti, Maria Ida De Michelis , Joerg Kudla , and Maria Cristina Bonza

Supplementary data

Fig. S1. Subcellular localisation of NUP::YC3.6 probe in a representative tobacco epidermal leaf cell.
Fig. S2. Comparison of wounding-induced Ca2+ transients in leaves of Nicotiana plants transiently transformed with NES::YC3.6 or NUP::YC3.6.
Fig. S3. Expression levels of PM-localised ACA isoforms in roots of Arabidopsis thaliana.
Video S1. Series of nuclear Ca2+ ratio images of a Nicotiana leaf subjected to wounding

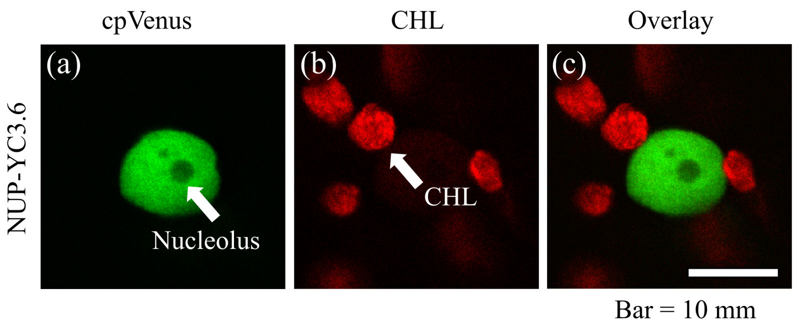

FIGURE S1

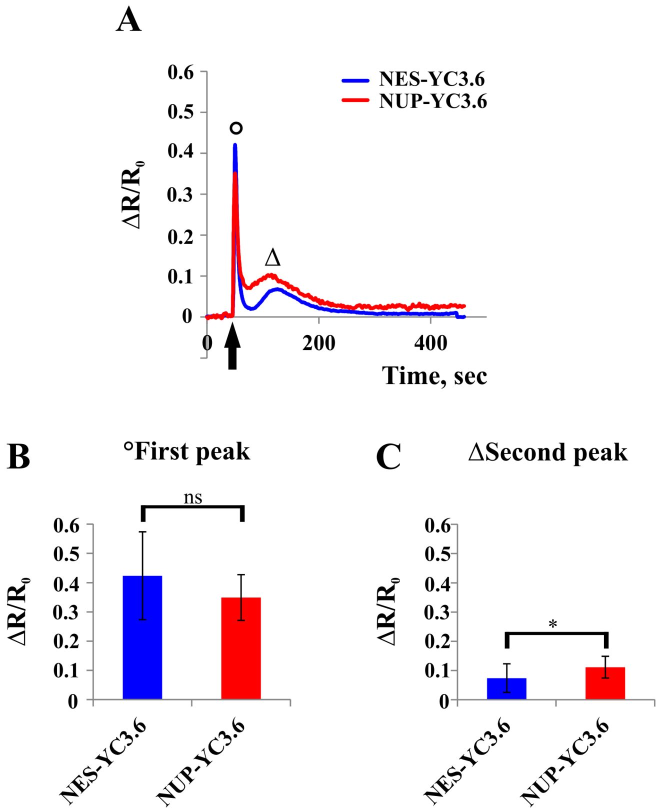
A
0.6
0.5
0.4
0.3
0.2
0.1
0
ΔR/R0
NES-YC3.6
NUP-YC3.6
o
Δ
0
200
400
Time, sec
B
°First peak
ns
0.6
0.5
0.4
0.3
0.2
0.1
0
ΔR/R0
NES-YC3.6
NUP-YC3.6
C
ΔSecond peak
*
0.6
0.5
0.4
0.3
0.2
0.1
0
ΔR/R0
NES-YC3.6
NUP-YC3.6

FIGURE S2

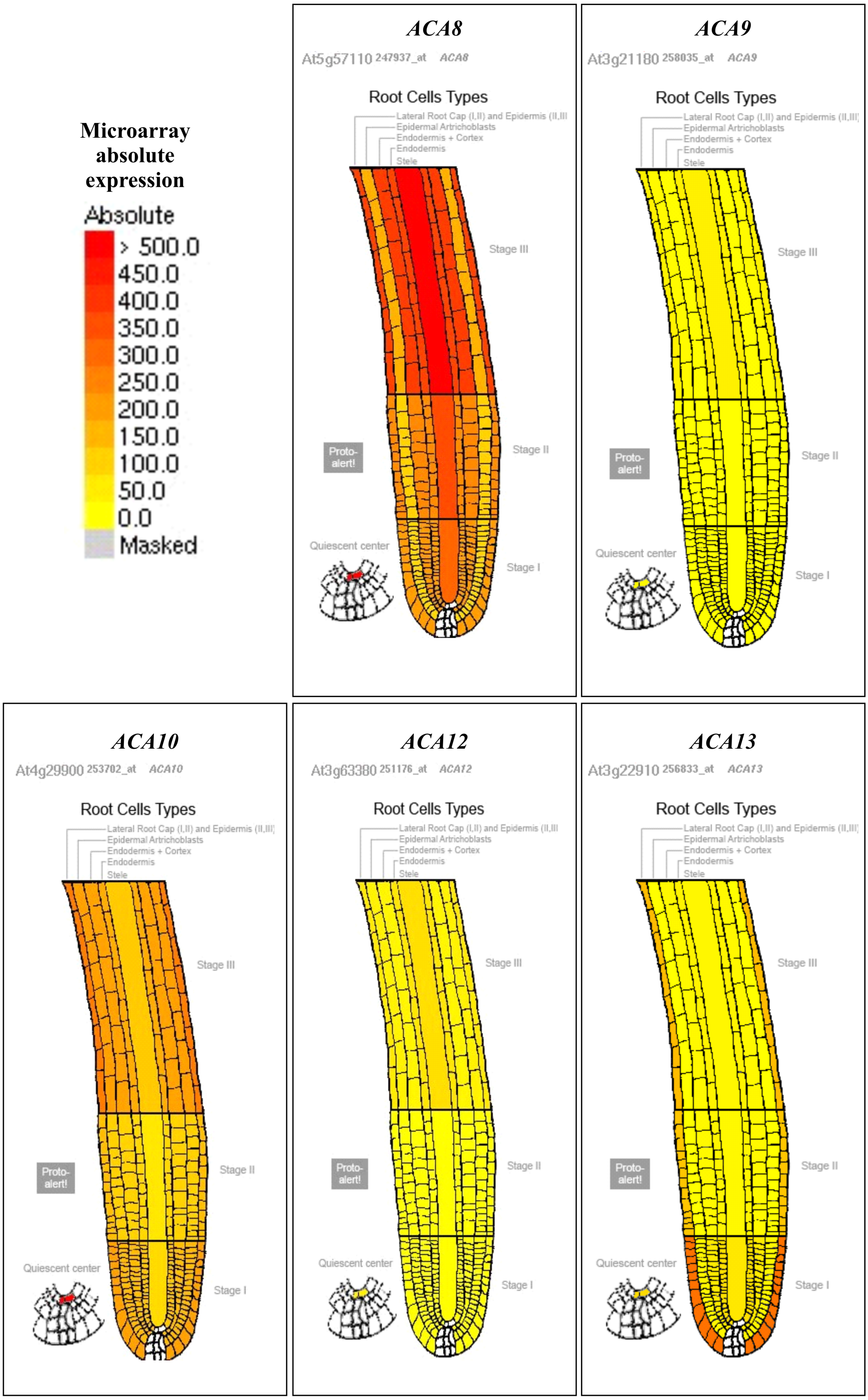
Microarray absolute expression
Absolute
> 500.0
450.0
400.0
350.0
300.0
250.0
200.0
150.0
100.0
50.0
0.0
Masked
ACA8
At5g57110 247937_at ACA8
ACA9
At3g21180 258035_at ACA9
ACA10
At4g29900 253702_at ACA10
ACA12
At3g63380 251176_at ACA12
ACA13
At3g22910 256833_at ACA13
Root Cells Types
Lateral Root Cap (I,II) and Epidermis (II,III)
Epidermal Artrichoblasts
Endodermis + Cortex
Endodermis
Stele
Stage III
Stage II
Stage I
Proto-alert!
Quiescent center

FIGURE S3
